# SSIM-Based Autoencoder Modeling to Defeat Adversarial Patch Attacks

**DOI:** 10.3390/s24196461

**Published:** 2024-10-06

**Authors:** Seungyeol Lee, Seongwoo Hong, Gwangyeol Kim, Jaecheol Ha

**Affiliations:** 1 Department of Information Security, Hoseo University, Asan 31499, Republic of Korea; stl990726@naver.com (S.L.); hshsw5660@gmail.com (S.H.); 2 Sinsiway Inc., Songpa-gu, Seoul 05836, Republic of Korea; jeffkim@sinsiway.com

**Keywords:** object detection, YOLO, adversarial patch attack, structural similarity index measure, autoencoder

## Abstract

Object detection systems are used in various fields such as autonomous vehicles and facial recognition. In particular, object detection using deep learning networks enables real-time processing in low-performance edge devices and can maintain high detection rates. However, edge devices that operate far from administrators are vulnerable to various physical attacks by malicious adversaries. In this paper, we implement a function for detecting traffic signs by using You Only Look Once (YOLO) as well as Faster-RCNN, which can be adopted by edge devices of autonomous vehicles. Then, assuming the role of a malicious attacker, we executed adversarial patch attacks with Adv-Patch and Dpatch. Trying to cause misdetection of traffic stop signs by using Adv-Patch and Dpatch, we confirmed the attacks can succeed with a high probability. To defeat these attacks, we propose an image reconstruction method using an autoencoder and the Structural Similarity Index Measure (SSIM). We confirm that the proposed method can sufficiently defend against an attack, attaining a mean Average Precision (mAP) of 91.46% even when two adversarial attacks are launched.

## 1. Introduction

Recently, object detection systems using deep learning networks have been used in autonomous vehicles, for face recognition, and in home networks. In particular, object detection that uses a deep learning network and runs on low-performance edge devices has the advantage of a real-time processing capability. Despite its performance, object detection is prone to adversarial attacks. In particular, it has been shown that adversarial patterns can be generated in the physical world, often taking the form of a patch. These physical adversarial patches pose a significant threat to edge devices where deep learning networks operate far from administrators. These attacks can compromise object detection systems that use deep learning networks, leading to accidents through misdetection in both autonomous vehicles and facial recognition.

Brown et al. [1] showed that an image classification model can fail with high probability if a patch is simply attached to an image, unlike existing evasion attacks in white-box and black-box environments. An adversarial patch attack targeting a traffic sign classifier was launched by Lengyel et al. [2]. Using adversarial patch attacks of various scales, they succeeded in classifying stop signs into different classes.

Since adversarial attacks on classifiers have been attempted, advanced attacks on object detectors have also been proposed. Thys et al. [3] executed adversarial patch (Adv-patch) attacks in both digital and real-world environments. They showed that detectors could not recognize people after an adversarial patch was attached to their bodies. Liu et al. [4] proposed Dpatch, an attack more advanced than a simple adversarial patch. Dpatch uses the peculiarities of bounding box regression and object classification in an object detection system and has a higher success rate than adversarial patches placed in various locations in the image.

Researching advanced patch generation, Eykholt et al. [5] launched an adversarial patch attack targeting a traffic sign classifier. The attack’s success rate was evaluated after masking the adversarial patch and setting up an environment that did not obscure traffic signs. Hu et al. [6] used a generative adversarial network to create an adversarial patch as natural as those in the real world while maintaining high attack performance.

In order to defeat adversarial patch attacks, Nasser et al. [7] demonstrated a Local Gradient Smoothing (LGS) method of responding to adversarial patch attacks by smoothing the high-frequency parts of images with an adversarial patch. However, this method, which relies heavily on thresholds, progresses toward smoothing the local elements of the image and incurs high computational costs, making it difficult to find optimal thresholds.

Furthermore, a method to counteract adversarial attacks by using an autoencoder was proposed by Yin et al. [8]. With an autoencoder, they countered attacks after removing noise from images. To address adversarial attacks, the authors employed an autoencoder to reconstruct images to prevent the reconstruction of malicious images. However, since additional memory modules are used, there is a disadvantage: the larger the size of the latent vector, the higher the calculation cost. The Segment and Complete defense (SAC) [9] adopts segment models to detect adversarial patches and feeds the masked images into the base object detector for prediction. However, it has the disadvantage of having difficulty removing an adversarial patch generated by unsupervised learning.

In this paper, we first implemented a function for detecting traffic objects such as stop signs and speed limit signs. We adopted the You Only Look Once (YOLO) and Faster-RCNN as deep learning object detection algorithms [10]. Then, assuming a malicious attacker, we launched Adv-Patch and Dpatch attacks targeting our algorithms. The original Adv-Patch and Dpatch attacks were successful, reducing the mean Average Precision(mAP) to about 22.16%.

To counter these attacks, we propose an image reconstruction method using an autoencoder and the Structural Similarity Index Measure (SSIM). The proposed method enables normal object detection by sending patched images to an autoencoder and reconstructing them into clean images without the need for memory modules and large computational costs. Additionally, the SSIM was used during the autoencoder training process to improve the quality of the reconstructed images.

The major contributions of our paper are as follows:

- We propose a novel defensive approach in traffic sign detection, which can suppress the effects of adversarial path attacks.
- We attempted adversarial patch attacks against YOLO and Faster-RCNN detectors using three traffic sign datasets.
- We perform experiments to evaluate whether the proposed SSIM-based autoencoder method is useful in the defense against adversarial patch attacks in traffic signs.
- The proposed defense method for traffic sign detection does not require large computational costs, and it retrains the weight-fixed object detector model.

This paper is structured as follows. Section 2 describes image reconstruction using autoencoders, adversarial patch attacks, Dpatch, and the SSIM. Section 3 explains adversarial patch attacks on object detectors. Section 4 explains image reconstruction using the proposed SSIM-based autoencoder. Section 5 describes the experimental environment and evaluates performance against adversarial patch attacks and Dpatch attacks by applying the proposed countermeasures. Section 6 concludes the paper.

## 2. Background

### 2.1. Adversarial Patch Attacks

Deep learning networks are used in many artificial intelligence industries but are exposed to several types of cyber attacks. In particular, they are vulnerable to evasion attacks that add noise to a network input image, which causes the deep learning network to malfunction. Representative adversarial evasion attacks include the Fast Gradient Sign Method (FGSM) [11], Projected Gradient Descent (PGD) [12], Carlini and Wagner (CW) [13], DeepFool [14] and the Jacobian-based Saliency Map Attack (JSMA) [15].

However, these types of attacks are difficult to try in the real world because they require the attacker to directly access the deep learning model. On the other hand, the adversarial patch attack attaches malicious patches to images without accessing the deep learning network, also causing malfunctions. Equation (1) is the formula for generating an adversarial patch [1,2,3]:

(1) $$\hat{p}=\arg\max\;\;\;E_{x∼X,t∼T,l∼L}\left[logPr\left(\hat{y}|A\left(p,x,l,t\right)\right)\right]$$

Here, *X* represents the input data. *T* is the distribution of transformations of the image, *L* denotes the distribution of patch locations within the image, $\hat{p}$ symbolizes the final patch, and $\hat{y}$ represents the intended class.

**Figure 1:**
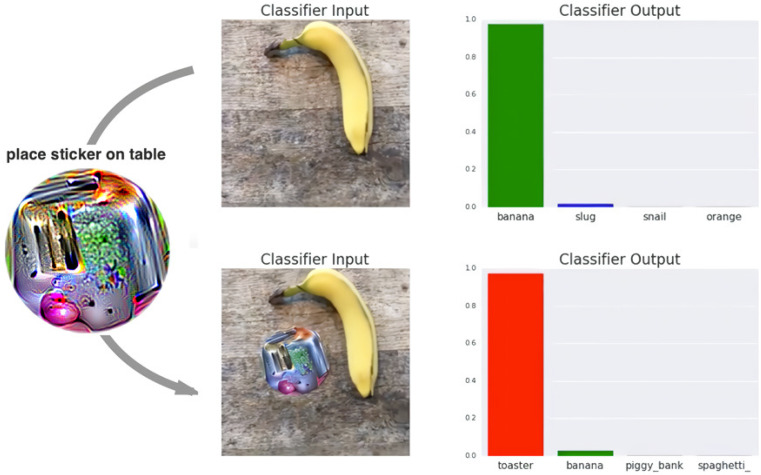
Successfully fooling a classification system by using an adversarial patch.

An attacker can cause a deep learning network to malfunction simply by attaching a generated adversarial patch to an image. Figure 1 shows an example of an adversarial patch attack where a classification model that was supposed to predict banana predicted toaster.

### 2.2. Dpatch

Dpatch is a patch-based adversarial attack technique to fool object detection models such as YOLO and Faster-RCNN [4]. Unlike the traditional adversarial patch, Dpatch is designed in a small form that can be physically attached to objects, causing object detection models to misclassify them or malfunction. Equation (2) is a formula generating a Dpatch [4]:

(2) $$\delta^{*}=argmax_{\delta }L\left(f\left(I+M\cdot \delta \right),y\right)$$

Here, *I* is the original image, δ refers to the patch, and *M* is the mask indicating the location of the patch. Additionally, *L* represents the loss function, and *y* is the actual label.

In a Dpatch attack, the patch is generated using gradient-based optimization. When the generated patch is attached to an object, it significantly reduces the performance of a deep learning network, which will eventually fail to correctly recognize objects like traffic signals.

### 2.3. Autoencoders

**Figure 2:**
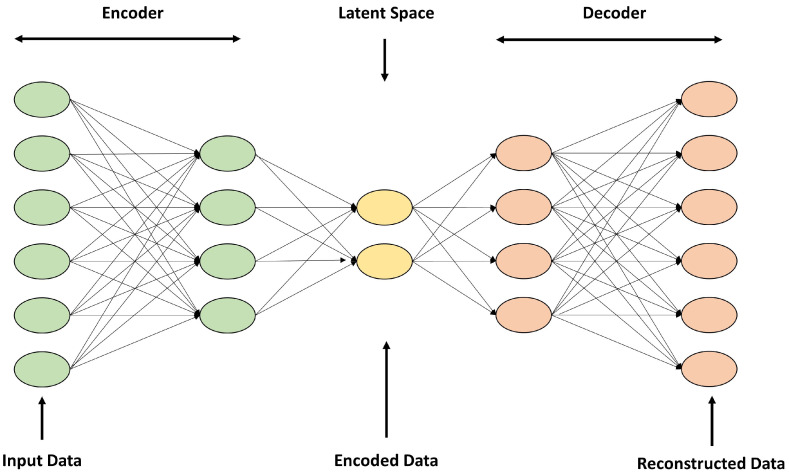
Structure of an autoencoder model.

An autoencoder is an unsupervised learning neural network that compresses and reconstructs input data. The autoencoder consists of an encoder, latent space, and a decoder, as shown in Figure 2. The encoder maps the input data into the latent space, and the decoder takes the compressed data mapped to the latent space and reconstructs the original data. Using these characteristics, autoencoders are used for data compression and image noise removal [16,17,18].

### 2.4. Structural Similarity Index Measure

SSIM is a method for assessing digital images and videos [19]. Unlike traditional methods to evaluate images, such as peak signal-to-noise ratio (PSNR), mean squared error (MSE), and Mean Absolute Error (MAE), SSIM evaluates image quality based on three main aspects: luminance, contrast, and structure.

Additionally, images reconstructed using SSIM may have higher super-resolution, which can be helpful in training image generation networks. Large SSIM values indicate more similarity to the original image, whereas small values indicate less similarity. Therefore, if you use SSIM during model training for image generation, you can reconstruct high-quality images. Equation (3) is the formula for calculating the SSIM value [19]:

(3) $$SSIM\left(x,y\right)={\left[l(x,y)\right]}^{\alpha }\cdot {\left[c(x,y)\right]}^{\beta }\cdot {\left[s(x,y)\right]}^{\gamma }$$

Here, *x* represents the original image, and *y* represents the generated image, while l,c, and *s* are luminance, contrast, and structure, respectively. The correlation coefficient between *x* and *y* is calculated using l,c, and s to evaluate similarity in an image, with α,β, and γ expressed in terms of the importance of *l*, *c*, and *s*, respectively.

## 3. Adversarial Patch Attacks on Object Detectors

### 3.1. Threat Model

We constructed a scenario where a malicious attacker induces a malfunction in the object detector. In this scenario, the attacker applies an adversarial patch to traffic signs, preventing the object detection model from recognizing them. A camera mounted on the vehicle captures a malicious patch that causes the object detection model to malfunction. These scenarios are extremely dangerous for autonomous vehicles, which rely on accurate object detection to drive safely. Furthermore, the adversarial patches could be deployed remotely or physically, increasing their potential threat level in real-world environments.

**Figure 3:**
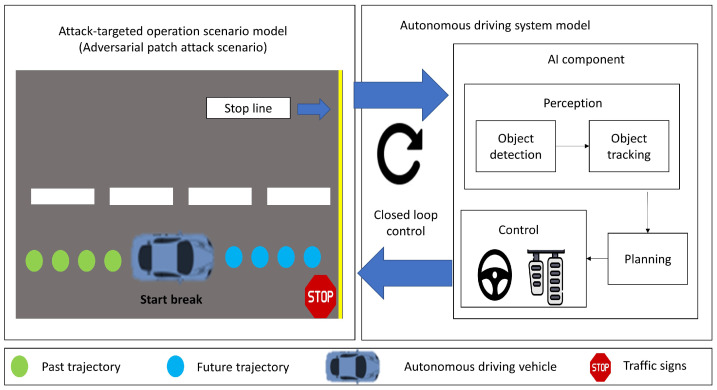
Object detection system model for adversarial patch attacks in autonomous driving car.

Figure 3 shows the aforementioned system model for the adversarial patch attacks. The autonomous driving system model for object detection, which is the targeted AI component in adversarial patch attacks, mainly includes its tasks of tracking, planning, and control, as well as closed loop control. The adversarial patch attacks scenario model, which includes speed limits and system-level attack goals, can lead to traffic rule violations such as hitting an object or crossing the stop line.

### 3.2. Adversarial Patch Attack Methodology

We executed adversarial attacks using the original Adv-Patch and Dpatch targeting YOLO versions that can detect traffic signs. Adv-Patch generation [3] from the original paper is based on decreasing object score loss, total variation loss, and non-printability score loss as predicted by the object detector.

First, the object score loss represents the score from an object detector that predicts an object in an image. Next, the total variation loss smooths the adversarial patch, reducing unnecessary noise in it. The formula for total variation loss is

(4) $$L_{tv}=\sum_{i,j}\sqrt{({\left(p_{i,j}-p_{i+1,j}\right)}^{2}+{\left(p_{i,j}-p_{i,j+1}\right)}^{2}}$$

Non-printability score loss is a value that guarantees the attack success rate when printing patches. Minimizing these losses allows adversarial patches to be used effectively in the real world. The formula for non-printability score loss is

(5) $$L_{nps}=\sum_{p_{patch\in p}}\min_{c_{print}\;\in \;C}\left|p_{patch}-c_{print}\right|$$

Our adversarial patch was generated by minimizing the object score loss for traffic signs in order to cause misdetection by YOLO while reducing total variation loss and non-printability score loss, resulting in the final adversarial patch. Additionally, the adversarial patch is resized and attached using a masking technique [20,21] to avoid obscuring the stop sign.

**Figure 4:**
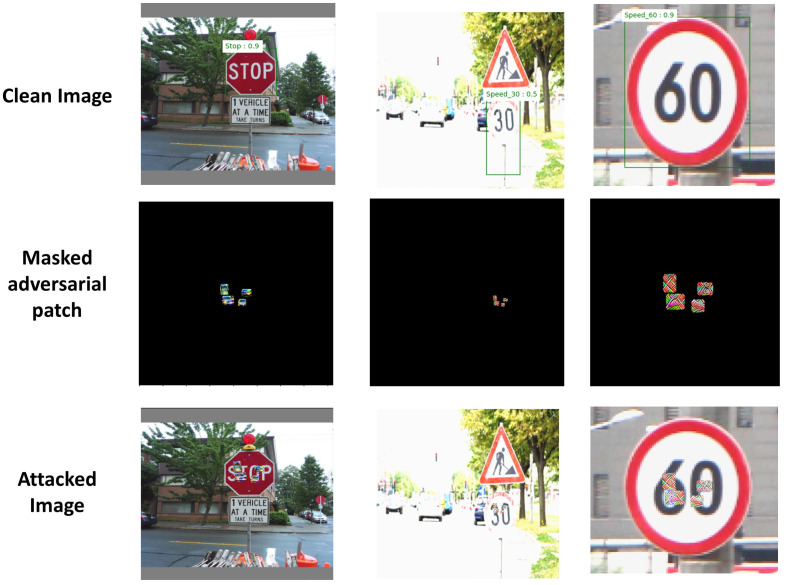
Adversarial patch attacks on traffic signs.

As shown in Figure 4, YOLO accurately detects traffic signs in clean images. We apply the detector first on images without a patch (top row), then with a masked patch (middle row), and then with our generated patch (bottom row). In most attacks, our patch was able to successfully hide traffic signs from the detector. However, we can catch the adversarial patch when it is not aligned with the center of the traffic sign. Therefore, for optimized attack success, the patch should be positioned in the center of the traffic sign as determined by the bounding box. We were able to generate adversarial patches according to the method described above [1,3], and confirmed that these patch attacks can be successful with a high probability.

## 4. SSIM-Based Autoencoder Modeling

**Figure 5:**
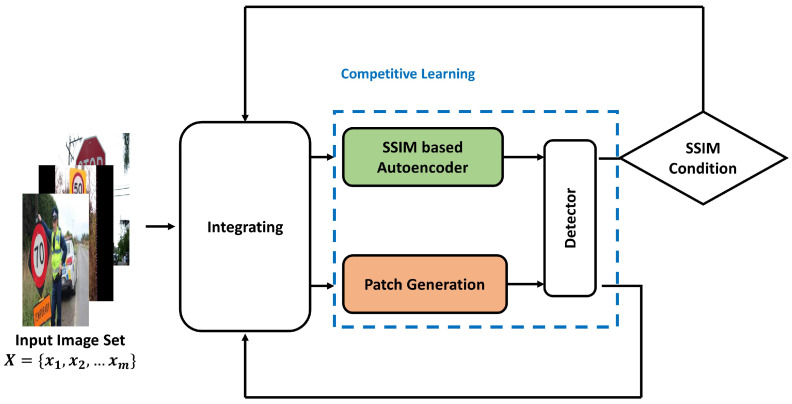
Competitive learning in the SSIM-based autoencoder model.

We developed a countermeasure against adversarial patch attacks that cause failures in object detectors. We propose a new competitive learning architecture using an SSIM-based autoencoder to defeat adversarial patch attacks, shown in Figure 5. This architecture consists of three components: a fixed-weight object detector such as YOLOv8 or Faster-RCNN, an SSIM-based autoencoder, and an adversarial patch generator. Here, the SSIM-based autoencoder created through competitive learning reconstructs an image with an adversarial patch into a clean image so that object detection is performed normally. This competitive learning allows the construction of a robust autoencoder that can withstand various adversarial patch attacks. Consequently, these two modules, the SSIM-based autoencoder and the patch generator, are designed to improve performance through mutual competition.

Furthermore, we use the SSIM method as an additional loss function to improve the quality of the generated clean image when the autoencoder reconstructs the adversarial patch image. By combining the SSIM method with an autoencoder, the final output image reconstructed through the autoencoder can be very close to a clean image.

The SSIM-based autoencoder generation module focuses on preventing traffic sign detection systems from malfunctioning due to adversarial patches. The SSIM evaluates the structural similarity of images, and based on this, the autoencoder reconstructs the input image into a normal image to make it similar to the original image. Generating autoencoder output similar to the input image means minimizing the impact of adversarial patch attacks.

The optimization process of the autoencoder is accomplished through the following objective function:

(6) $$\Delta AE=\nabla_{AE}L_{AE}\left(x,g\left(f\left(L_{patch}\left(x,p\right)\right)\right)\right)+1-SSIM\left(x,g\left(f\left(L_{patch}\left(x,p\right)\right)\right)\right)$$

Here, *x* represents the input image, and *f* and *g* are the encoder and decoder, respectively. $L_{patch}$ denotes a function that attaches a patch, *p*, to *x*.

The process for modeling the SSIM-based autoencoder is shown in Algorithm 1. The adversarial patch generation module reduces the object score from traffic sign detection by optimizing adversarial patch *p*. This process is described in Algorithm 1 as $\Delta p=\nabla_{p}L_{patch}\left(x,p\right)$. The patch generation module updates patch *p* for each input data point *x* and calculates the gradient of patch *p*. In this process, a patch that interferes with traffic sign detection is created by ensuring that the detector’s object score is 0.

The computation in Line 6 $\Delta p=\nabla_{p}L_{patch}\left(x,p\right)$ is the process of updating adversarial patch *p* in the initial learning stages, and Line 8 in the later stages, $\Delta p=\nabla_{p}L_{patch}\left(x,p,f,g\right)$, updates the patch more powerfully. Here, competitive learning parameters $E,K_{p}$ and $K_{AE}$ from Algorithm 1 to train the SSIM-based autoencoder are set to 3, 5, and 5, respectively.

These two modules (the SSIM-based autoencoder and the patch generator) complement each other through competitive learning. The SSIM-based autoencoder tries to maintain the original detection performance, while the adversarial patch generation module tries to interfere with it. In this process, the autoencoder iteratively learns to minimize the impact of adversarial patches by reconstructing images containing adversarial patches into normal images.

The SSIM-based autoencoder provides generalized defense performance against a variety of attacks as well as specific types of adversarial patches. The SSIM described in Line 14 of Algorithm 1 evaluates the structural similarity of two images: the normal image and the reconstructed one generated by the autoencoder. Here, the SSIM value is always less than 1, with higher values indicating more similarity. This competitive learning approach can minimize the effect of adversarial patches and significantly improves the stability of a traffic sign detection system.
**Algorithm 1:** SSIM-based Autoencoder Modeling**Input:** Data point *X***Output:** AE1:Initialize $p_{0}\leftarrow$ Gaussian Noise2:**repeat**                  ▹ Iterative learning process3:    **for** each data point x∈X **do**       ▹ Process of patch generator4:        **for** $k=1,2,\ldots ,K_{p}$ **do**5:           **if** E==1 **then**6:               $\Delta p=\nabla_{p}L_{patch}\left(x,p\right)$7:           **else**8:               $\Delta p=\nabla_{p}L_{patch}\left(x,p,f,g\right)$9:           **end if**10:        **end for**11:    **end for**12:    **for** each data point x∈X **do**       ▹ Process of autoencoder13:        **for** $k=1,2,\ldots ,K_{AE}$ **do**14:           $\Delta AE=\nabla_{AE}L_{AE}\left(x,g\left(f\left(L_{patch}\left(x,p\right)\right)\right)\right)+1-SSIM\left(x,g\left(f\left(L_{patch}\left(x,p\right)\right)\right)\right)$15:        **end for**16:    **end for**17:**until** training epoch *E*

## 5. The Countermeasure against Adversarial Patch Attacks

### 5.1. Traffic Sign Detection in Normal Detector

We used YOLOv8 and Faster-RCNN object detection models for the adversarial patch attacks targeting traffic signs after pre-training them on the COCO dataset [22]. Before proceeding with the experiment, 958 images containing stop signs were pulled. From them, 674 images were used for competitive training with the SSIM-based autoencoder. After modeling the SSIM-based autoencoder, we evaluated the performance of object detection models YOLOv8 and Faster-RCNN. Here, we use 284 testing images with stop signs from the COCO dataset. In the LISA dataset [23], we evaluate performance using 300 stop sign images from 1034 training images. In addition, after learning YOLOv8 and Faster-RCNN from the Traffic Sign dataset [24] in a similar way, we also evaluate the detection performance on 158 images containing the speed limit class. The dataset used in our experiments is shown in Table 1.

To calculate performance by the YOLOv8 and Faster-RCNN, the minimum detection threshold was set to 0.5. Intersection over Union was set to 0.65, and images were sized at 416 × 416. For the attacks that disabled object detection, we chose the original Adv-Patch and Dpatch because they are popular attack methods for assessing vulnerabilities in object detection models.

To generate an adversarial patch, we used 674 images on the COCO dataset and 729 images on the Traffic Sign dataset. The patches were created with a size of 320 × 320, and after applying masking, they were attached to traffic signs. We used Adam to optimize patch generation. After applying masking to each attack with an initial learning rate of 0.03, we attached patches to traffic signs. Here, the optimization was performed for a total of 300 epochs.

To generate an adversarial patch, YOLOv8 used class loss and objectness loss as the loss functions, while the Faster R-CNN model used classifier loss, box regression loss, objectness loss, and RPN box regression loss as the loss functions. The size of the patch was adjusted to a scale value of 0.45, taking into account the size of the object. While increasing the scale value can improve the attack success rate, it has the disadvantage of obscuring the object.

For the LGS countermeasure [7], we set the block size to 15 and the overlap to 5. The thresholds were set to 0.1 for Faster-RCNN and 0.25 for the YOLOv8. And then the smoothing factor was designed as 1.8 Faster-RCNN, 5.5 for YOLOv8.

For the AE countermeasure [8], we used 674 images form COCO dataset and 792 images from the Traffic Sign dataset, using input images resized to 416 × 416, with a batch size of 12 and 100 epochs. We use Adam optimizer with a learning rate of 0.005 for our gradient descent with a ReduceLROnPlateau scheduler. Here, we chose the loss functions as Mean Squared Error(MSE). The latent space dimensionality was set to 26.

For the SAC countermeasure [9], the pre-training Unet model on the COCO dataset was adopted with the base filter being 16. Here, square size, the size of an adversarial patch, was used 125, 100, 75, 50, or 25.

Furthermore, we evaluated the detection model performance by varying brightness and contrast in the images to create an environment similar to real traffic. The brightness value was randomized from 0 to 3, where 1 indicates no effect, 0 indicates darkening, and closer to 3 means brighter. The contrast value was randomized from 0.7 to 1.3, where 1 is no impact, 0.7 is decreased contrast, and 1.3 is increased contrast. Table 1 shows the object detection performance of the YOLO model on clean images from the COCO, LISA, and Traffic Sign datasets. As seen in Table 2, object detection was achieved with a maximum mean Average Precision (mAP) of 98.88% and a minimum of 96.71% using the YOLOv8 detector.

### 5.2. Results on Adversarial Patch Attacks

We assumed the use of an adversarial patch in a real-world situation. This means that many factors affected the appearance of a patch from a patch attack. The lighting can change, or the size of the patch with respect to the traffic sign can change; the input sensor might add noise or might blur the patch slightly. To take a real environment into account as much as possible, we made the following random transformations on the patches before applying them to the original images: (1) The patch was scaled up or down randomly; (2) Random noise was added to the patch; (3) Brightness and contrast were changed randomly.

Table 3 shows the YOLOv8 and Faster-RCNN performance under adversarial patch attacks when using the COCO, LISA, and Traffic Sign datasets. From the three datasets, minimum mAP scores for the YOLOv8 were 26.55% in an Adv-patch attack on the Traffic Sign dataset. Additionally, Faster-RCNN showed mAP scores of 54.36% from an Adv-patch attack and 42.96% from Dpatch. Launching two adversarial attacks on all the datasets confirmed that most mAP scores decreased significantly, making it impossible to detect objects. Specifically, we found that adversarial attacks against YOLOv8, which showed the maximum detection rate when not attacked, dropped to 26.55% in the worst case scenario.

### 5.3. Object Detection Using Proposed SSIM-Based Autoencoder

**Figure 6:**
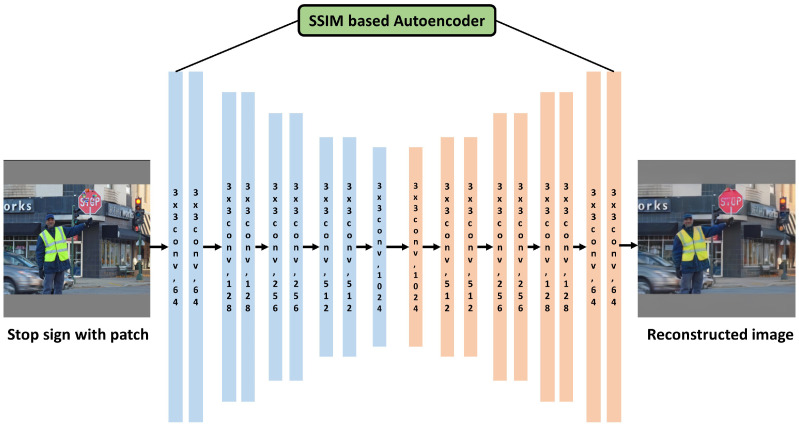
The SSIM-based autoencoder architecture.

In order to defeat these adversarial attacks, we propose an image reconstruction method using an SSIM-based autoencoder. We made the autoencoder model in order to reconstruct stop sign images containing adversarial patches into clean images and to improve the quality of the reconstructed images through the SSIM. Thereafter, the object detector could identify image input from the SSIM-based autoencoder normally. We improved the robustness of the SSIM-based autoencoder by using competitive learning to reconstruct images with different adversarial patches. Figure 6 shows the SSIM-based autoencoder architecture to counteract these adversarial patch attacks.

**Figure 7:**
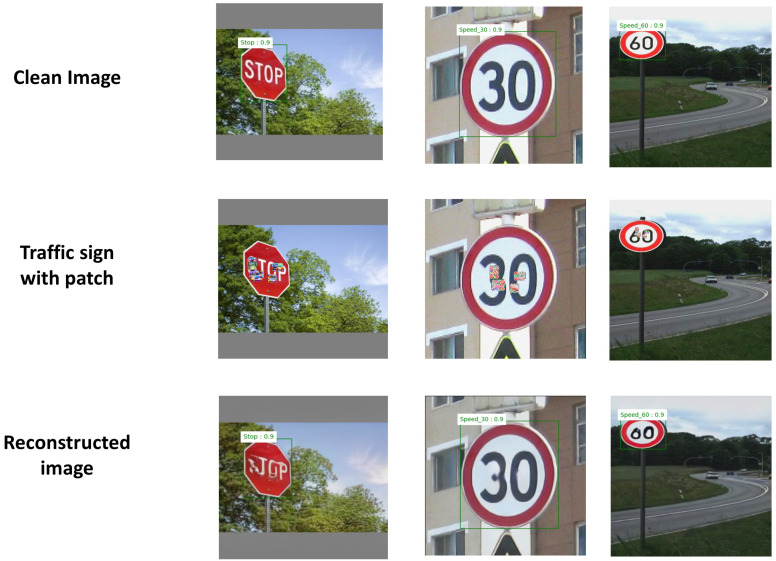
Traffic sign images reconstructed with the SSIM-based autoencoder.

Examples of detection using the SSIM-based autoencoder on images of traffic signs are shown in Figure 7. The top images are clean, and the middle images have patches generated by an attack. The bottom images were reconstructed by the SSIM-based autoencoder.

Table 4 shows the experimental results from three datasets and two types of detection models. According to Table 4, for clean images without adversarial attacks, the mAP scores of the YOLOv8 and Faster-RCNN reached 98.85%.

When launching adversarial patch attacks on the YOLOv8, mAP decreased to 26.55% under the Adv-Patch attack and to 39.86% from Dpatch (see Table 3). Table 4 shows the proposed SSIM-based autoencoder improved mAP to 92.88% and 95.19% despite the Adv-Patch and Dpatch attacks, respectively. Additionally, we obtained similar results by using the SSIM-based autoencoder on Faster-RCNN. As shown in Table 4, mAP improved to 92.75% and 90.79% for the Adv-Patch and Dpatch attacks, respectively.

The proposed SSIM-based autoencoder can maintain performance comparable to a detector in the absence of adversarial attacks without any meaningful degradation. In particular, accuracy under the Adv-Patch attack against YOLOv8 dropped to 26.55% at worst (see Table 3). However, using the autoencoder proposed in this paper, we were able to detect 91.56% of the attacks (see Table 4). This is slightly lower than the performance without an attack, but was the best performance among the detectors. In addition, the detection rate of YOLOv8 was 4.54% and 7.31% higher than under adversarial attacks compared to Faster-RCNN, confirming it is an attack-resistant detector. Consequently, experimental results demonstrated that the proposed autoencoder can significantly defend against adversarial patch attacks and can be applied to both single-stage and two-stage detection networks.

## 6. Conclusions

Recently, object detection based on deep learning has been widely used in autonomous vehicles, facial recognition, and smart factories. However, these object detection systems are vulnerable to the adversarial patch attacks that can be carried out without access to a detector. Even a simple patch attack can cause an object detection system to miss an object, which can lead to a serious accident.

In this paper, we implemented traffic stop sign detection by using three versions of the YOLOv8 and Faster-RCNN in edge devices, and we launched adversarial patch attacks using Adv-Patch and Dpatch. The experiment confirmed that the YOLO family’s mAP decreased to 26.55% under the original Adv-patch attack and to 39.86% under the Dpatch attack. Additionally, mAP for Faster-RCNN decreased to 54.36% under the Adv-Patch attack and to 42.96% under the Dpatch attack.

By adding the SSIM to an autoencoder, we attained a countermeasure from a deep learning model that reconstructs an attacked image into a clean image and improves the detection rate of traffic signs. Our experiment with the YOLOv8 detector confirmed that the proposed SSIM-based autoencoder was able to restore the mAP of the object detector to 91.46%. This confirmed that the proposed deep learning model is effective against adversarial patch attacks compared to image reconstruction methods such as the original autoencoder, LGS, and SAC. Therefore, we need to build more training datasets in real traffic environments and develop novel detection models that can defeat patch attacks, which can be the direction of future work.

## Figures and Tables

**Table 1:** Training and testing datasets of traffic signs.

Dataset	Training Datasets	Testing Dataset
COCO Dataset (Stop signs)	674	284
LISA Dataset (Stop signs)	1034	300
Traffic Sign Dataset (Stop and speed signs)	729	158

**Table 2:** Object detection using COCO, LISA, and Traffic Sign datasets.

Dataset	YOLOv8	Faster-RCNN
COCO Dataset (Stop signs)	98.03%	96.05%
LISA Dataset (Stop signs)	96.71%	98.55%
Traffic Sign Dataset (Stop and speed signs)	98.88%	96.16%

**Table 3:** Performance on detection models applied adversarial patch attacks.

Dataset	Attack Method	YOLOv8	Faster-RCNN
COCO Dataset	Adv_Patch [3]	73.48%	53.38%
	Dpatch [4]	65.45%	41.28%
LISA Dataset	Adv_Patch [3]	49.16%	49.05%
	Dpatch [4]	40.45%	22.16%
Traffic Sign Dataset	Adv_Patch [3]	26.55%	54.36%
	Dpatch [4]	39.86%	42.96%

**Table 4:** Evaluation of performance of SSIM-based autoencoder with competitive learning.

Dataset	Attack Method	YOLOv8								Faster-RCNN							
		Ours		LGS [7]		AE [8]		SAC [9]		Ours		LGS [7]		AE [8]		SAC [9]	
		mAP	Recall	mAP	Recall	mAP	Recall	mAP	Recall	mAP	Recall	mAP	Recall	mAP	Recall	mAP	Recall
COCO Dataset	No attack	97.68%	98.59%	95.99%	98.24%	87.41%	92.61%	98.03%	99.30%	88.60%	89.77%	94.63%	96.16%	79.65%	80.19%	96.05%	97.12%
	Adv-patch [3]	94.93%	95.77%	77.79%	82.39%	80.77%	80.63%	78.34%	79.87%	86.23%	87.54%	76.31%	79.87%	75.50%	72.20%	46.73%	58.46%
	Dpatch [4]	94.98%	95.07%	88.40%	82.39%	82.22%	83.10%	64.40%	68.66%	85.08%	86.90%	69.87%	62.32%	71.88%	71.56%	60.33%	64.85%
LISA Dataset	No attack	96.65%	98.33%	95.58%	97.33%	93.82%	97.33%	96.71%	97.33%	95.07%	95.68%	95.29%	97.01%	90.95%	89.03%	98.55%	98.33%
	Adv-patch [3]	92.88%	94.00%	78.34%	73.00%	90.14%	91.33%	77.65%	77.67%	92.75%	94.35%	66.65%	68.43%	88.40%	87.70%	20.84%	36.54%
	Dpatch [4]	95.19%	95.07%	88.93%	82.39%	87.19%	83.10%	48.13%	49.33%	90.79%	93.68%	65.85%	82.06%	82.65%	85.71%	37.44%	53.48%
Traffic Sign Dataset	No attack	98.85%	98.95%	97.27%	98.31%	80.18%	100%	98.88%	100%	96.16%	96.25%	96.50%	96.47%	81.81%	83.37%	96.16%	96.29%
	Adv-patch [3]	91.56%	93.46%	71.65%	79.08%	63.54%	71.09%	38.05%	60.38%	87.02%	91.91%	52.02%	67.92%	43.64%	56.30%	24.54%	42.05%
	Dpatch [4]	91.46%	90.86%	74.98%	85.81%	67.63%	90.86%	35.33%	59.10%	84.15%	88.42%	43.95%	59.29%	43.32%	52.76%	33.81%	43.45%

## Data Availability

Data are contained within the article.
